# Thermal performance of gasifier cooking stoves: A systematic literature review

**DOI:** 10.12688/f1000research.126890.2

**Published:** 2023-07-05

**Authors:** Md Insiat Islam Rabby, Md Wasi Uddin, Mahafuzur Rahman Sheikh, Humayun Kabir Bhuiyan, Tazeen Afrin Mumu, Fabliha Islam, Afsana Sultana

**Affiliations:** 1 Department of Mechanical Engineering, Military Institute of Science and Technology, Mirpur, Dhaka, 1200, Bangladesh; 2 Faculty of Engineering, Universiti Putra Malaysia, Selangor, Malaysia

**Keywords:** Gasified cooking stoves, thermal performance, cooking fuels and literature review.

## Abstract

A systematic literature review was conducted to summarize the overall thermal performance of different gasified cooking stoves from the available literature. For this purpose, available studies from the last 14 years (2008 to 2022) were searched using different search strings. After screening, a total of 28 articles were selected for this literature review. Scopus, Google Scholar, and Web of Science databases were used as search strings by applying “Gasifier cooking stove” AND “producer gas cooking stove” AND “thermal performance” keywords. This review uncovers different gasified cooking stoves, cooking fuels, and fabrication materials besides overall thermal performances. The result shows that the overall thermal performance of different gasified cooking stoves was 5.88% to 91% depending on the design and burning fuels. The premixed producer gas burner with a swirl vane stove provided the highest overall thermal performance range, which was 84% to 91%, and the updraft gasified stove provided the lowest performance, which was 5.88% to 8.79%. The result also demonstrates that the wood pellets cooking fuel provided the highest thermal performance and corn straw briquette fuel provided the lowest for gasified cooking stoves. The overall thermal performance of wood pellets was 38.5% and corn straw briquette was 10.86%.

## Introduction

One of the largest energy-consuming sectors in developing nations is the cooking sector, and this sector requires a large amount of energy and effort as it is a commonplace daily activity. Biomass fuel, natural gas, oil, and coal are the predominant sources of energy for cooking sector, and the majority of the inhabitants in developing countries rely on conventional fuels, typically wood and agricultural residues. Approximately three billion people worldwide, 41% of households, rely on solid biomass fuels (biomass such as wood, crop residues, animal waste, charcoal, and coal) for cooking due to the affordability or availability of these fuels, especially in developing countries in Asia and sub-Saharan Africa ( Bonjour *et al.*, 2013). The majorities of the conventional cooking are perpetrated over open flames, which burn inefficiently and result in significant emissions. It is worth to be mentioned that, in 2010, about 3.5 million premature deaths globally were caused by household air pollution ( Lim *et al.*, 2012), and it also contributed to outdoor air pollution, which resulted in an additional 370,000 deaths and 9.9 million disability-adjusted life worldwide ( Chafe *et al.*, 2014). Furthermore, household emissions can stimulate lung cancer, chronic obstructive pulmonary disease and chronic bronchitis, cardiovascular diseases, low birth weight, stillbirth, and acute lower respiratory infections ( Amegah & Jaakkola, 2016). Excessive uses of solid fuels have pernicious effects on human health, regional environment, and global climate ( Smith *et al.*, 2004). Due to the pernicious impact on human health that results in sophisticated diseases, global temperature rise, hazardous gas emissions, and excessive time waste in conventional cooking, the advancement of heat generation techniques in cooking stoves become significant.

To concoct an improved cooing stove, it must requires substantial improvements in combustion efficiency as well as increased fuel efficiency compared to conventional stoves ( Venkataraman *et al.*, 2010). In the first decade of the 1940s, the development of biomass-based cooking stoves commenced in India, and these stoves were known as improved mud cooking stoves. Then another study ( Raju, 1954) reported the development of the upgraded multi-pot mud cooking stoves for Indian rural households. Afterwards, an upsurge in better cooking stoves appeared due to the world's focus shifted to environmental concerns and energy conservation measures. These cooking stoves were created and built using engineering principles, making them more effective and long-lasting than the conventional open fired cooking stove. Investigators are currently attempting to design cooking stoves that are more ecologic and sustainable as well as more energy and thermally efficient. To date, several different types of improved cooking stoves have been designed and investigated, i.e. patsari cooking stoves ( Cynthia *et al.*, 2008), mirt cook stove ( Dresen *et al.*, 2014), gasifier cook stove ( Carter *et al.*, 2014), wick stove ( Dinesha *et al.*, 2019), pellet stoves ( Boman *et al.*, 2011), radiant stoves ( Pantangi *et al.*, 2011), etc. From the above verities, gasifier cook stove is one of the potential energy efficient and environment friendly cook stove.

The process of transforming solid or liquid feed stocks into usable gaseous or other chemical fuels that may be combusted to produce thermal energy is known as gasification. Fuel with a small amount of air is delivered into a closed container so that the fuel can be partially combusted to generate the required heat for gasification. The fundamental idea of gasification is that it is a thermochemical process that uses the reactions of drying, pyrolysis, oxidation, and reduction to turn solid fuel into a combustible gas (producer gas) ( Basu, 2010). In a gasifier cook stove, biomass is gasified in the reactor to generate syngas, thereafter, syngas is burned in the burner in order to obtain producer gas flame ( Susastriawan *et al.*, 2021). On the contrary, biomass is directly combusted with the presence of excess air and produced heat and flue gas.

Due to the eclectic amount of highly appealing characteristics of gasifier cook stoves, including high efficiency, smoke-free safe combustion, uniform and steady flame, simplicity of controlling the flame, and operational capability for long periods ( Raman *et al.*, 2014), the advancement of gasifier cooking stoves became significant. Therefore, to date, several research studies had been performed on the design and development of gasifier cooking stoves with the goal of increasing efficiency and dwindling emission such as producer gas stove with bluff-body shape in burner ( Susastriawan *et al.*, 2021), producer gas stove ( Panwar *et al.*, 2011; Punnarapong *et al.*, 2017), Chinese gasifier stove ( Carter *et al.*, 2014), natural-draft gasifier cook stoves ( Hailu, 2022; Tryner *et al.*, 2014), fixed bed advanced micro-gasifier cook stove ( Sakthivadivel & Iniyan, 2017), inverted downdraft gasifier ( Narnaware & Pareek, 2016; Ojolo *et al.*, 2012; Osei *et al.*, 2020), biomass gasifier cook stove ( Panwar & Rathore, 2015), top-lit updraft gasifier cook stove ( Scharler et al., 2021), advance micro-gasifier stove ( Sakthivadivel *et al.*, 2019; Wamalwa *et al.*, 2017), rice husk gas stove ( Ndindeng *et al.*, 2019), natural and force draft gasifiers stove ( Getahun *et al.*, 2018), and natural cross draft ( Nwakaire & Ugwuishiwu, 2015). However, to the authors’ best knowledge, no proper systematic reviews have already been conducted on the overall thermal performance of gasifier cook stoves, with an emphasis on types of gasifier stoves, cooking fuels, location of investigation, and materials to fabricate stoves. Therefore, in this study, a systematic review has been performed to consolidate all the technical works published on the thermal performance of gasifier cooking stoves as well as further analyse the areas on which additional studies should be focused for future research trajectory.

## Methods

**Figure 1.**
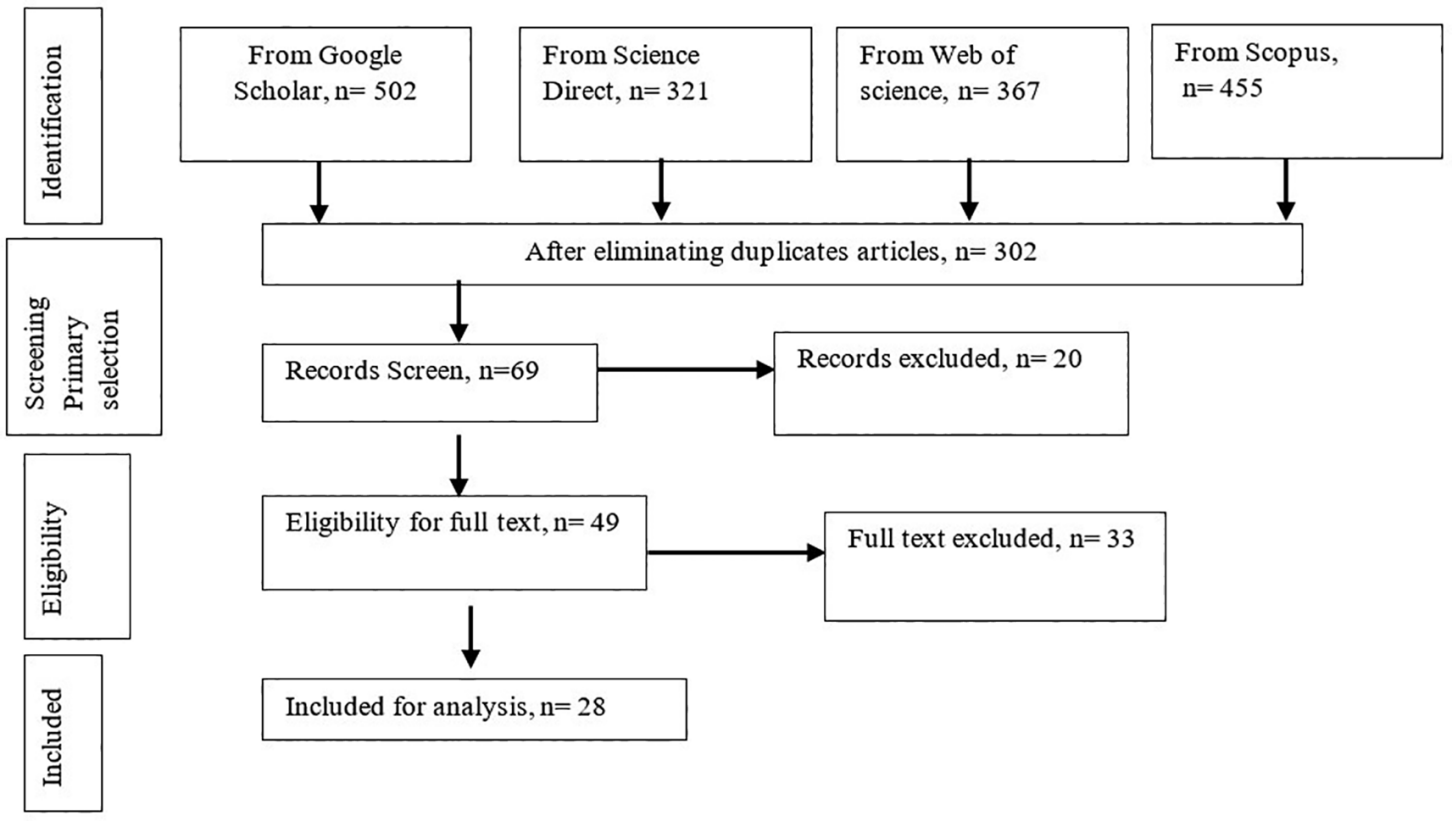
Summary of study selection design.

A typical research methodology steps for systematic review of Tranfield *et al.* (2003) are considered which are given in Figure 1 wherein the 1 ^st^ stage is known as “Define” which is subdivided by steps as “Identification of need for a literature review” and “Development of a literature review protocol”. The 2 ^nd^ stage known as “Collect and Select” which is also consist of two steps- “Identification of documents” and “Selection of relevant documents”. Simultaneously, the 3 ^rd^ stage is “Analyse” which is categorized as documents and Data extraction steps. Meanwhile, the final stage is “Result” indicates the last steps “Documents Finding” wherein collected all documentation are reviewed significantly for extracting knowledge from gathered information.

### Search and selection strategy

A literature search was conducted to cover the period from January 2008 to August 2022. Scopus, Web of Science, Google Scholar and Science Direct databases were selected as search strings. Boolean operators “AND” and “OR” between Keywords and database searching fields. The searching keywords were gasified cooking stoves, producer gas cooking stove thermal performance and cooking fuels. The gasified cooking stove also called as producer gas cooking stove therefore both of the terms used as keywords. EndNote X 9.0 software was used to exclude duplicates from searched data. The protocol of the review discussed in Table 1.

**Table 1.** Protocol of review.

Items	Descriptions
Keywords	Gasifier cooking stove, producer gas cooking stove, thermal performance and emission of pollutants
Boolean Operators	“AND” between Keywords; “OR” between Database search fields.
Search Fields	Abstract; Title; Keywords;
Exclusion Criteria	Household survey study, review article, articles that did not determine thermal performance or emission of pollutants
Language	English
Publication Type	Article
Time Window	January 2008 to August 2022
Searching Keywords	Gasified cooking stoves, producer gas cooking stove thermal performance, cooking fuels

### Data extraction and analysis

To conduct this study, the author, date, name and types of study, study location, stoves types, material used, fuels/energy sources, thermal performances and emission of pollutants were reported by using Microsoft Excel.

## Results and discussion

A total of 1153 articles initially identified. After removing duplicates, checking title, abstract and full text, 28 were found eligible based on the predetermined exclusion and inclusion criteria for this study. Among the 28 selected articles, all conducted their investigation on gasified cooking stoves experimentally and only 3 articles performed numerical/computational analysis beside experimental study.

### Publication year

**Figure 2.**
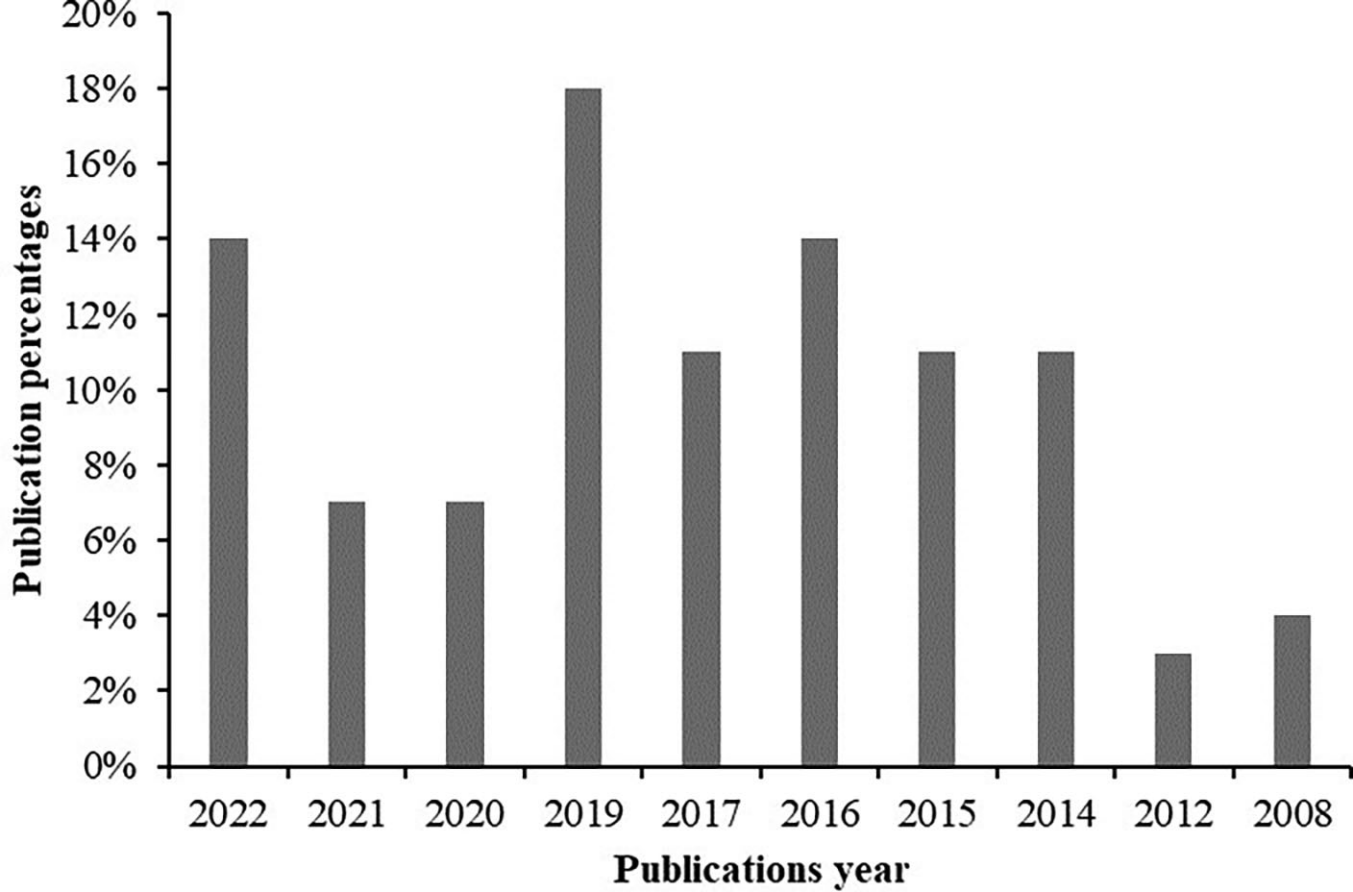
Year wise publications percentages.

The publications year of the selected articles is summarized in Figure 2, which was obtained from Table 2. The figure shows that the selected articles were published in 2022, 2021, 2020, 2019, 2017, 2016, 2015, 2014, 2012 and 2008. The result also highlights that the highest amount of research on gasified cooking stoves was conducted in 2019 at 18% and the lowest amount of research was conducted in 2012 at only 4%. From the beginning to the mid of the current year 2022 almost 14% studies were identified from the selected literature which reflects that the investigation demand on gasified cooking stoves is recently also a high priority to researchers.

**Table 2.** Summary of findings reported in selected articles.

Authors	Gasified stove types	Study types	Study locations	Material used	Fuel/energy source	Thermal efficiency
Hailu, 2022	Natural draft	Experimental	Not mentioned	Steel sheet or cast iron	Eucalyptus, bamboo, and sawdust-cow animal waste briquettes	29.85%, 28.43% and 23.76% for eucalyptus, sawdust-cow animal waste briquette, and bamboo
Himanshu *et al.*, 2022	Forced draft with separate secondary and primary air fans	Experimental and computational	India	Not mentioned	Biomass pellets	41-43%
Gutiérrez *et al.*, 2022	General gasified	Experimental	Not mentioned	Not mentioned	Wood pellets and wood chips from pine patula as fuels;	25.2% for pellets and 24.1% for chips.
Varunkumar *et al.*, 2012	General gasified	Experimental and computational	Not mentioned	Not mentioned	Wood	45– 47%
Scharler *et al.*, 2021	Top-lit updraft	Experiment and Numerical	Not mentioned	Not mentioned	Wood pellets and rice hull pellets	42%
Susastriawan *et al.*, 2021	Producer gas stove with bluff-body shape in burner	Experimental	Indonesia	Mild Steel	Rice husk and sawdust wastes	17.6%
Andika and Nelwan, 2020	Updraft	Experimental	Not mentioned	Carbon steel for chamber and ceramic wool for insulation	Cassava peel	5.88 to 8.79%
Osei *et al.*, 2020	Inverted downdraft	Experimental	Ghana	Stainless steel	Rice husk	30.5-38.1%
Ahmad *et al.*, 2019	Top-lit updraft (TLUD) with remote burner and fuel reactor	Experimental	China	Not mentioned	Peanut shell pellets, corn cobs, wood chips	31.4±1.2 for peanut shell pellets, 27.1±0.9% for corn cobs and 23.3±0.7% for wood chips.
Desale, 2019	Forced draft	Experimental	India	Stainless steel	Neem stalk	36.47%
Getahun *et al.*, 2018	Natural and forced draft	Experimental	Ethiopia	Not mentioned	Charcoal	22.7% and 25% for natural draft and forced draft respectively.
Ndindeng *et al.*, 2019	Rua rice husk stove (RRHS), Viet rice husk stove (VRHS), Paul Oliver 150 rice husk stove (PO150), Paul Olivier 250 rice husk stove (PO250) and Mayon rice husk gasifier stove (MYN)	Experimental	Sub-sahara africa	Stainless steel and cast iron	Rice husk	11% for MYN gasifier while 30% for PO150 and 20% for other stoves.
Sakthivadivel *et al.*, 2019	Advanced micro	Experimental	Not mentioned	Carbon steel	Coconut shells, tamarind pellet and Prosopis juliflora	36.7 ± 0.4%, 37 ± 0.4% and 38 ± 0.4%, for coconut shells, Prosopis juliflora and tamarind seed pellets, respectively.
Punnarapong *et al.*, 2017	Premixed producer gas burner with a swirl vane	Experimental	Thailand	Steel sheet and Ceramic fiber	Charcoal	84 – 91%
Sakthivadivel and Iniyan, 2017	Fixed bed advanced micro	Experiment	India	Carbon steel	Biomass fuels like coconut shells, prosopis Juliflora and wood pellets	36.7%, 36% and 38.5% for coconut shell, Prosopis Juliflora and wood pellets, respectively
Wamalwa *et al.*, 2017	Micro	Experimental	Kenya	Not mentioned	Saw dust pellets	36%
Ahmad *et al.*, 2016	Top lit up-draft (TLUD)	Experimental	China	Not mentioned	Wood char, rice husk, corn cob, nut shell pellets and corn straw briquette	17.8%, 16.47%, 14.38%, 12.38% and 10.86% for woodchar, rice husk, corncob, and nut shell pellets and corn straw briquette, respectively.
Chen *et al.*, 2016	Chinese three forced-draft	Experimental	China	Not mentioned	Pellets made with cornstalk and cow animal waste	16% to 43%
Narnaware and Pareek, 2016	Downdraft	Experimental	Not mentioned	Mild steel	Mango (magnifera indica), babul (prosopis julifera) and nim (azadirachta indica) wood	36 to 39%,
Panwar and Rathore, 2015	General gasified	Experimental	India	Mild steel	Biomass (Prosopis juliflora)	36.38%
Balakumar *et al.*, 2015	Forced draft micro	Experimental	India	Not mentioned	Juliflora wood and Coconut shell	for high power hot and cold start 28% and 30% for coconut shell and 27% and 28% for juliflora wood.
Kumar *et al.*, 2015	Chinese model (HX-20) updraft institutional	Experimental	Nepal	Not mentioned	Wood chips, rice husk and pellet	17.76%, 15.51% and 19.91% for wood chips, rice husk and pellet
Nwakaire and Ugwuishiwu, 2015	Natural cross draft	Experimental	Sub-saharan africa	Mild steel	Rice husk briquette	21.10%
Carter *et al.*, 2014	Chinese general gasified	Experimental	China	Not mentioned	Processed (pelletized) biomass	22 to 33%.
Shahi *et al.*, 2014	General gasified	Experimental	Nepal	Not mentioned	Pinusroxburgii (Salla) wood	34%
Tryner *et al.*, 2014	Natural-draft semi	Experiment	Not mentioned	Steel sheet	Corn cobs and wood pellets	42%
Ojolo *et al.*, 2012	Inverted downdraft	Experimental	Nigeria	Not mentioned	Biomass wood shaving	10.6%.
Panwar and Rathore, 2008	General gasified	Experimental	India	Mild steel	Babul wood and gas	26.5%

### Identified gasified cooking stoves

From the literature search, this review identified different types of gasified cooking stoves wherein modification and improvement were applied. Based on the findings from Table 2 the identified gasified cooking stoves are summarized in few categories, which are:

1. General gasified cooking stove: Biomass, Chinese, and biochar stoves.
2. Updraft gasifier cook stove: Updraft gasifier, Top-Lit Up Draft (TLUD) gasifier, portable TLUD gasifier, TLUD with remote burner and fuel reactor, reverse-downdraft, inverted downdraft, Chinese model (HX-20) updraft institutional.
3. Downdraft gasified stove: Downdraft gasifier and biomass downdraft.
4. Natural draft gasified stove: Natural draft and natural cross draft.
5. Forced draft gasified stove: Forced draft, forced draft pellet-fed semi gasifier, and forced draft with separate secondary and primary air fans.
6. Micro gasified stove: Fixed bed advanced micro and advanced micro.
7. Others: Producer gas stove with bluff-body shape in burner, rice husk gasifier stove, etc.

From the table it can be seen that most of the studies worked on general gasified cooking stoves while lowest number of studies worked on micro, and other gasified cooking stoves. Due to the easy design consideration and fabrication, most of the studies considered general gasified cooking stoves for their investigation. A short description of the categorized cooking stoves are as follows:

### General gasified cooking stove

**Figure 3.**
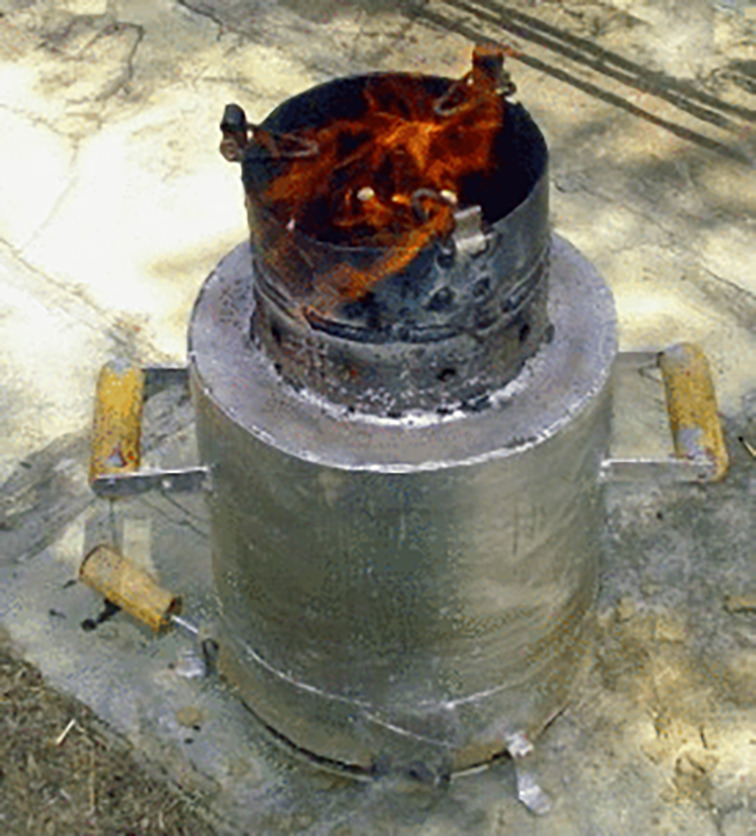
Bio-char general gasified cooking stoves ( Shahi *et al.*, 2014).

Few published articles have focused on gasified cooking stoves, but have not mentioned any particular type. These stoves are generally referred to as 'general gasified cooking stoves' in the literature. Biomass, Chinese, and biochar are identified as general gasified cooking stoves in literature. A bio-char general gasified cooking stove of Shahi *et al.* (2014) is presented in Figure 3. The stove mainly consists of outer cylinder ad inner cylinder. Inside the inner cylinder the combustion and then gasification occurs. The working procedure of these stoves depend on two processes. Firstly, charcoal and hydrocarbon-containing gases are combined with solid biomass in the gasification process. Second, a clear (smokeless) flame is used to burn the gases. At this stage, the stove's operation is halted when making charcoal, and the charcoal is taken out as a residue. For gasification, a primary air flow is necessary, and to help the gas ignite, a secondary air flow is added to the hot gas above the fuel.

### Updraft gasifier cook stove

Updraft gasifier cook stoves are a type of biomass stove that produce a clean and efficient flame through a process of partial combustion and gasification of the fuel. The basic principle of the updraft gasifier cook stove is to burn the fuel in an oxygen-limited environment, creating a syngas consisting of hydrogen, carbon monoxide, and other combustible gases. This gas is then burned cleanly in a secondary combustion chamber, producing a hot and efficient flame.

**Figure 4.**
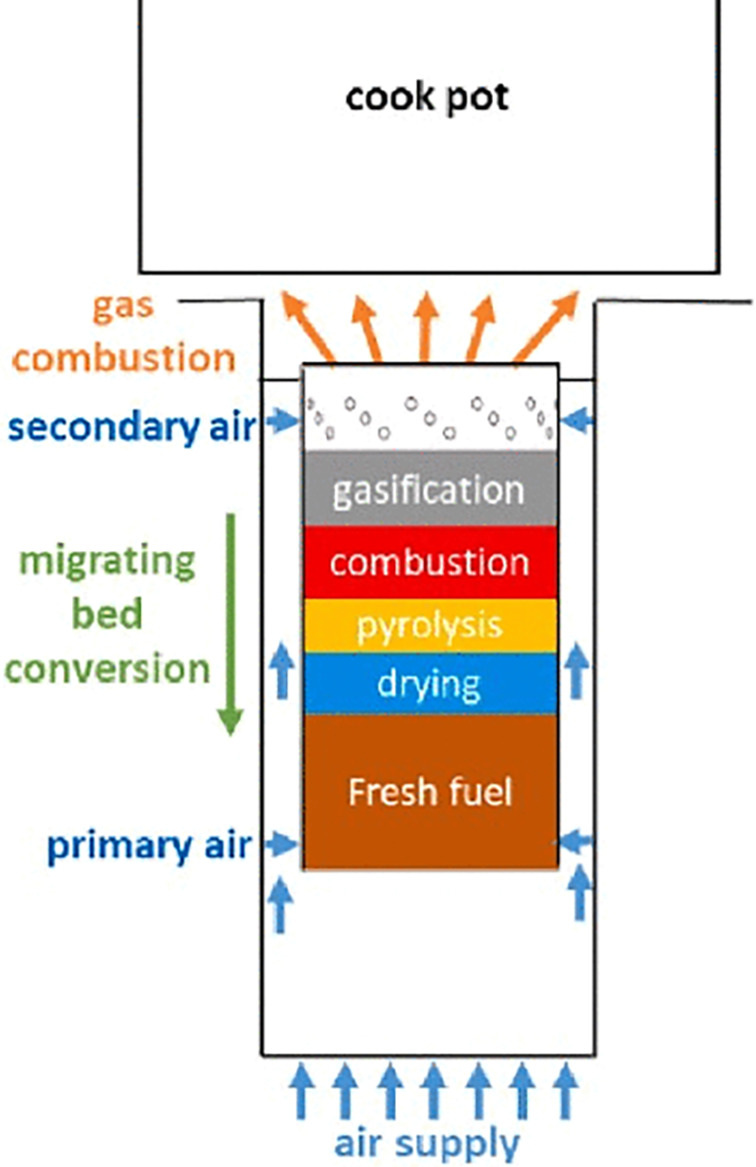
Schematic diagram of a Top-Lit Up Draft (TLUD) gasifier cook stove ( Scharler *et al.*, 2021).

Figure 4 illustrates the schematic diagram of a Top-Lit Up Draft (TLUD) gasifier cook stove ( Scharler *et al.*, 2021). The TLUD, known as the reverse downdraft gasifier, is a highly popular cook stove technology due to its ease of use and flexibility. It offers the same level of adaptability as the updraft gasifier, but with the added advantage of the downdraft gasifier: volatiles, including tar, produced during pyrolysis are partially decomposed and burned as they pass through the hot char bed above. This TLUD, as shown in Figure 4, enhances its flame efficiency by utilizing external fans or blowers. However, the TLUD stove can also be used by natural draft flow ( Tryner *et al.*, 2014).

### Downdraft gasified stove

A downdraft gasified stove is a type of cooking stove that operates by burning wood or other biomass in a closed chamber, which produces a gas that is then burned in a secondary combustion chamber to generate heat. The downdraft design of the stove allows for more efficient and complete combustion, resulting in lower emissions and higher energy efficiency compared to traditional open fire cooking.

**Figure 5.**
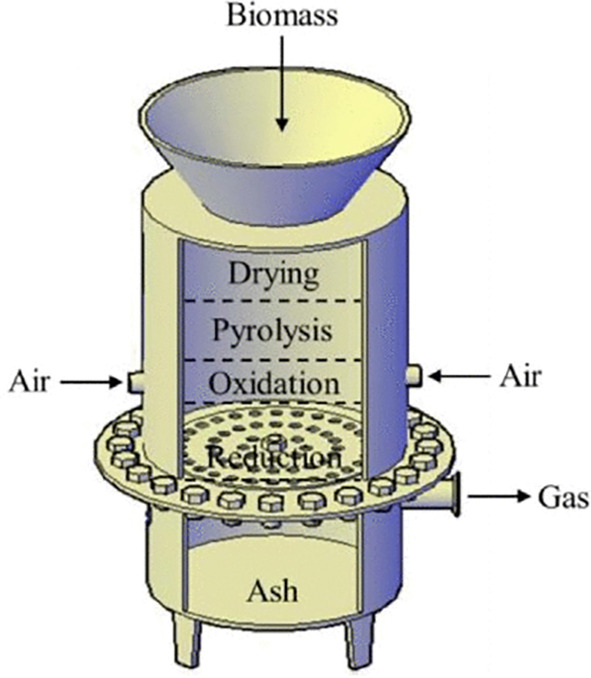
Schematic diagram of downdraft gasifier ( Susastriawan & Saptoadi, 2017).

The downdraft gasifier involves introducing biomass feedstock into the top of the gasifier, where it undergoes a series of processes including drying, pyrolysis, oxidation, and reduction as it moves downwards through the gasifier, as depicted in Figure 5 ( Susastriawan & Saptoadi, 2017). The gasification process produces a gas called producer gas which exits the gasifier through an outlet at the bottom. Producer gas is typically composed of both combustible gases, including CO, H _2_, and CH _4_, and non-combustible gases like CO _2_ and N _2_.

### Natural draft gasified stove

**Figure 6.**
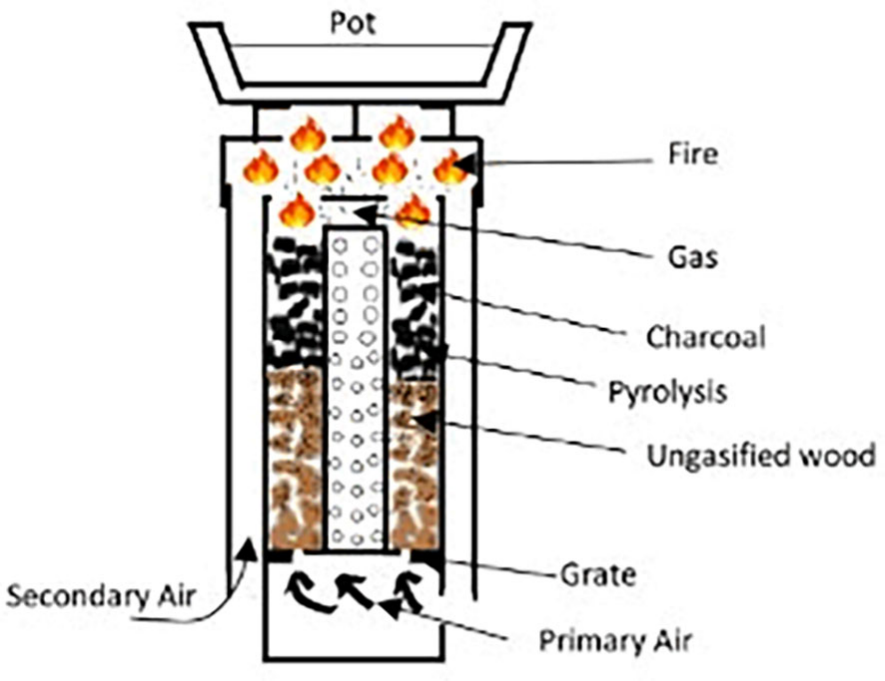
Natural draft gasified stove ( Hailu, 2022).

The gasifier stoves are built from sheet metals using basic mechanical techniques, and they include a fuel chamber for loading biomass residue, air inlets for partial combustion, and a pot stand for holding cooking utensils. This particular gasifier stove is natural, so it does not require any external power source to drive the primary and secondary air into the stove, unlike other gasifier stoves that rely on electricity. Figure 6 illustrates a schematic diagram of natural up draft gasifier stove. This stove is constructed by Hailu (2022) from mild steel sheet metal and can hold a maximum of 0.0005 m ^3^ (500 gm) of fuel for effective gasification. Secondary air enters the stove through the gap between the external cylinder and the internal gasifier chamber. The study suggests that the heat generated by the gasifier chamber's surface plays a crucial role in the combustion process. Specifically, this heat warms up the secondary air by means of conduction and convection, creating optimal conditions for combustion at the top of the gasifier chambers exterior. Due to this efficient process, the gasifier is able to function effectively and produce the desired output.

### Forced draft gasified stove

A forced draft gasified stove is a type of cook stove that uses a fan to introduce air into the combustion chamber at a higher pressure than the surrounding air. This results in a more efficient combustion process, with fuel burned more completely and at a higher temperature. Forced draft stoves also often include features such as insulation and preheated air supplies, which further optimize the combustion process and minimize heat loss.

**Figure 7.**
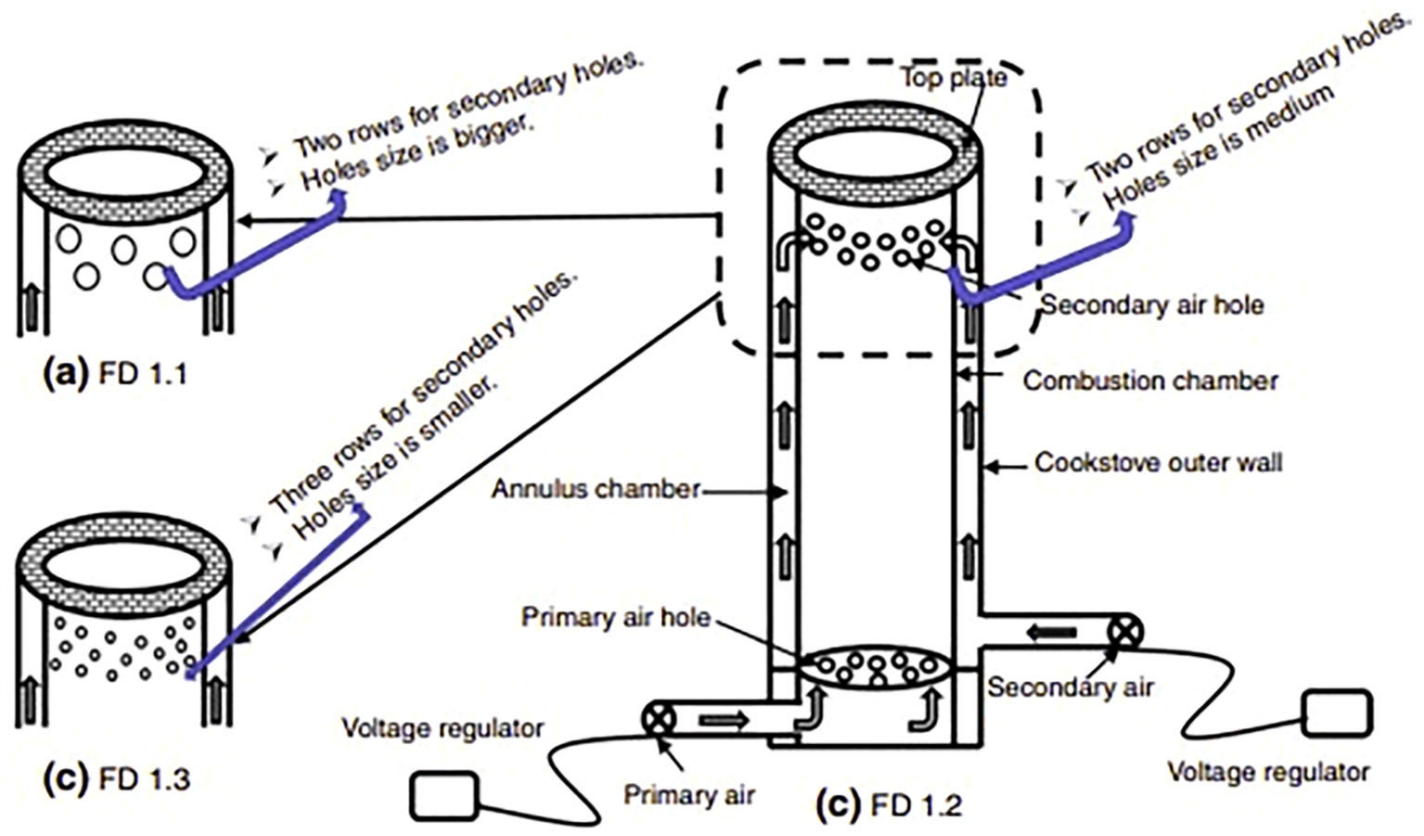
A forced draft gasified stove with two separate fans to supply primary and secondary air ( Himanshu *et al.*, 2022).

Figure 7 depicts a forced draft gasified stove with two separate fans to supply primary and secondary air ( Himanshu *et al.*, 2022). According to their study, the primary air was fed from a grate located below the fuel bed, which facilitated gasification, while the secondary air was introduced from the top of the cook stove and utilized to burn the volatiles released during biomass pellet gasification. The design also included an annulus chamber that preheated the secondary air before it entered the combustion chamber, minimizing heat loss and leading to a more efficient, cleaner combustion process. Overall, the study found that these measures were highly effective in optimizing the cook stove's performance. By providing a steady supply of preheated air and facilitating optimal gasification and combustion processes, the cook stove was able to produce the desired results while minimizing waste and reducing its environmental impact.

Forced draft gasified stoves have the potential to greatly reduce fuel consumption and minimize indoor air pollution, particularly in developing countries where traditional cooking methods can be both inefficient and harmful to human health. Further research and development in this area may lead to even more effective and sustainable cook stove technologies.

### Micro gasified stove

**Figure 8.**
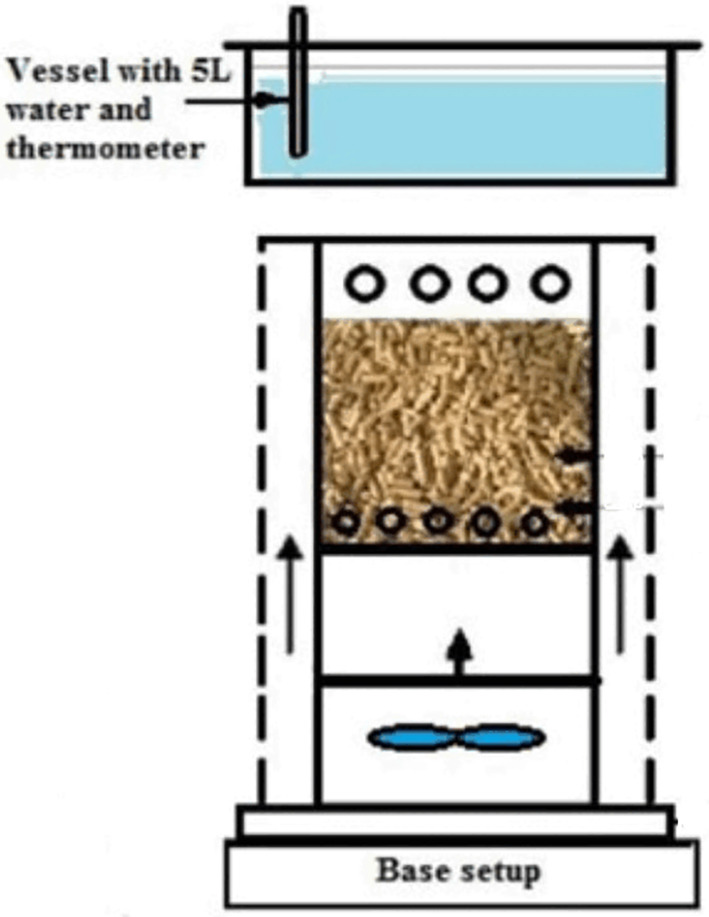
Advanced micro-gasifier cook stove ( Sakthivadivel & Iniyan, 2017).

A micro gasified stove is a small and portable stove that converts solid biomass fuel into a clean-burning gas. Figure 8 illustrates an experimental setup of an advanced micro-gasifier cook stove, built by Sakthivadivel and Iniyan (2017). The working principle of a micro gasified stove involves the partial combustion of solid biomass fuel in a low-oxygen environment. As the fuel heats up, it releases volatile gases, which are then burned in a separate combustion chamber to produce a clean-burning gas. This gas can be used to cook food or heat water, providing a convenient and efficient source of energy. The velocity of the air determines the rate of flame propagation of biomass fuel in fixed bed micro-gasifiers. The combustion air velocity, combustion process, and heat transfer all have an impact on the flame propagation, and are influenced by various fuel properties such as size, density, thermal conductivity, moisture content, ash content, and calorific value. Additionally, parameters such as bed porosity, peak temperature of the combustion chamber, and heat losses from the reactor can also affect flame propagation.

### Investigation location

**Figure 9.**
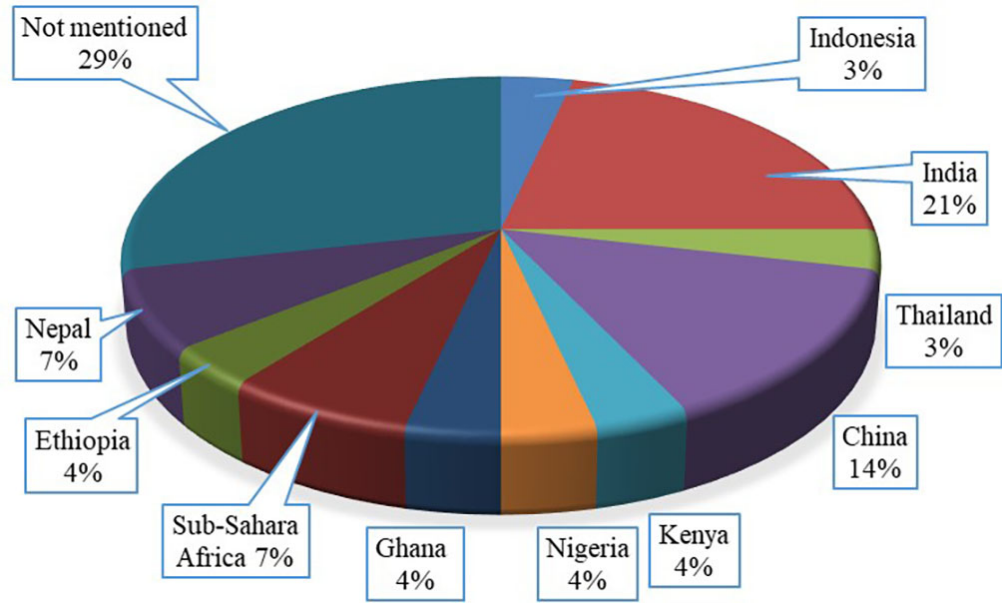
Country wise publication percentages.

Most of the identified articles on different gasified gas stoves are conducted in Asian and African continents as due to energy security and crisis people in these continents for which people of these continents mainly depend on the biomass fuel driven cooking system. Country wise identified published articles from Table 2 are presented in Figure 9. Among the selected articles 71% mentioned their study location. The figure shows 11 different countries from Asian and African continent where the investigation on gasified cooking stoves were conducted. The figure also highlights that 21% published articles performed their studies in India, which is the highest while the lowest study was performed in Thailand, which was only 3%. The design, configuration and burning fuels for any cooking stoves usually develop and investigate based on the geographical locations, climate, environment and materials availability. Therefore, this finding may help researchers, organizations and government to investigate and implement this type of cooking stoves based on the geographical location so that the adoption rate of the research can increase.

### Materials to fabricate stoves

Cast iron, mild steel, metal, ceramic fiber, steel sheet, carbon steel and stainless steel were mainly used to manufacture the gasified cooking stoves. Among the selected articles for the current review, only 60% articles addressed the materials they used to fabricate their experimental gasified stove. From Table 2 it can be said that various types of steel were the main materials for manufacturing the body of the gasified cooking stoves among those mild steel was applied mostly. The availability of mild steel in the investigated locations and higher thermal properties of stainless steel for cooking devices are the key reasons for applying it in production. In some studies, cast iron was used with steel for manufacturing purposes due to the cost effectiveness of cast iron and better mechanical wear resistance property. Additionally, ceramic fiber and wool were used for insulation purposes for updraft type and Premixed producer gas burner with a swirl vane type gasified cooking stoves.

### Cooking fuels

**Figure 10.**
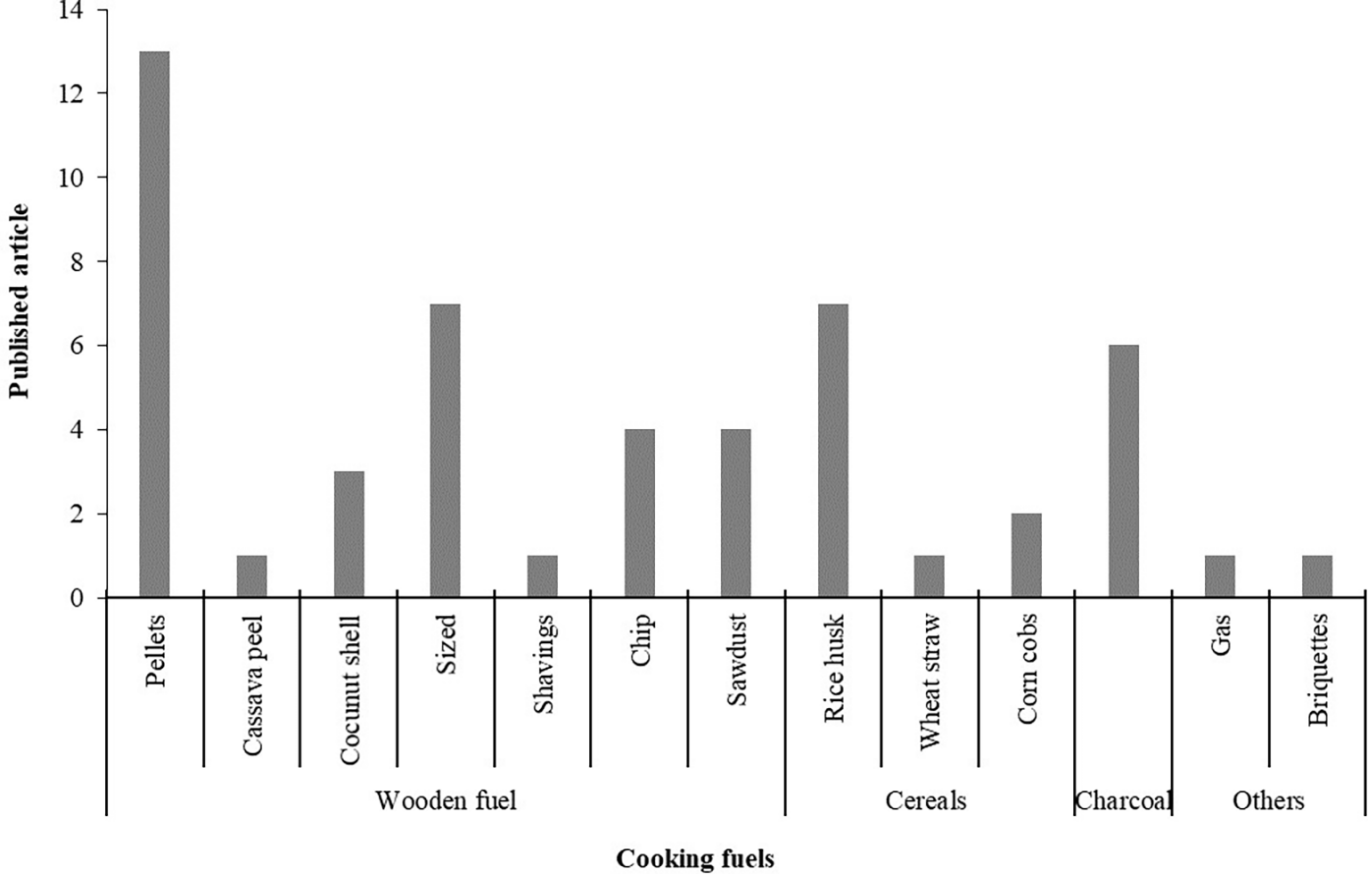
Cooking fuels based on published articles.

The fuels used in the cooking stoves are categorized in four types from the Table 2 and presented in Figure 10. The categories are wooden fuel, animals manure, cereals, charcoal and others. However, wooden fuels are classified in seven types, which are pellets, cassava peel, coconut shell, sized, shavings, chip and sawdust. Among the fuels wooden pellets fuels were used maximum. Peanut shell, cornstalk and cow animal waste, from pine patula, saw dust pellets, tamarind pellet, wood pellets and rice hull pellets are identified as wooden pellets fuels from selected articles. Moreover, Babul wood (Prosopis Juliflora), mango (magnifera indica), babul (prosopis julifera) and nim (azadirachta indica) wood, eucalyptus, bamboo and pinusroxburgii (Salla) wood are identified as sized wooden fuels. The rice husk, wheat straw and corncobs are categorized as cereal fuels while gas and briquettes are categorized in other types. In briquette fuels rice husk, sawdust-cow animal waste and corn straw are identified. This finding highlights the potential fuels to run a gasified cooking stove through which general people and research will be benefited.

### Thermal performance of different gasified cooking stoves

**Figure 11.**
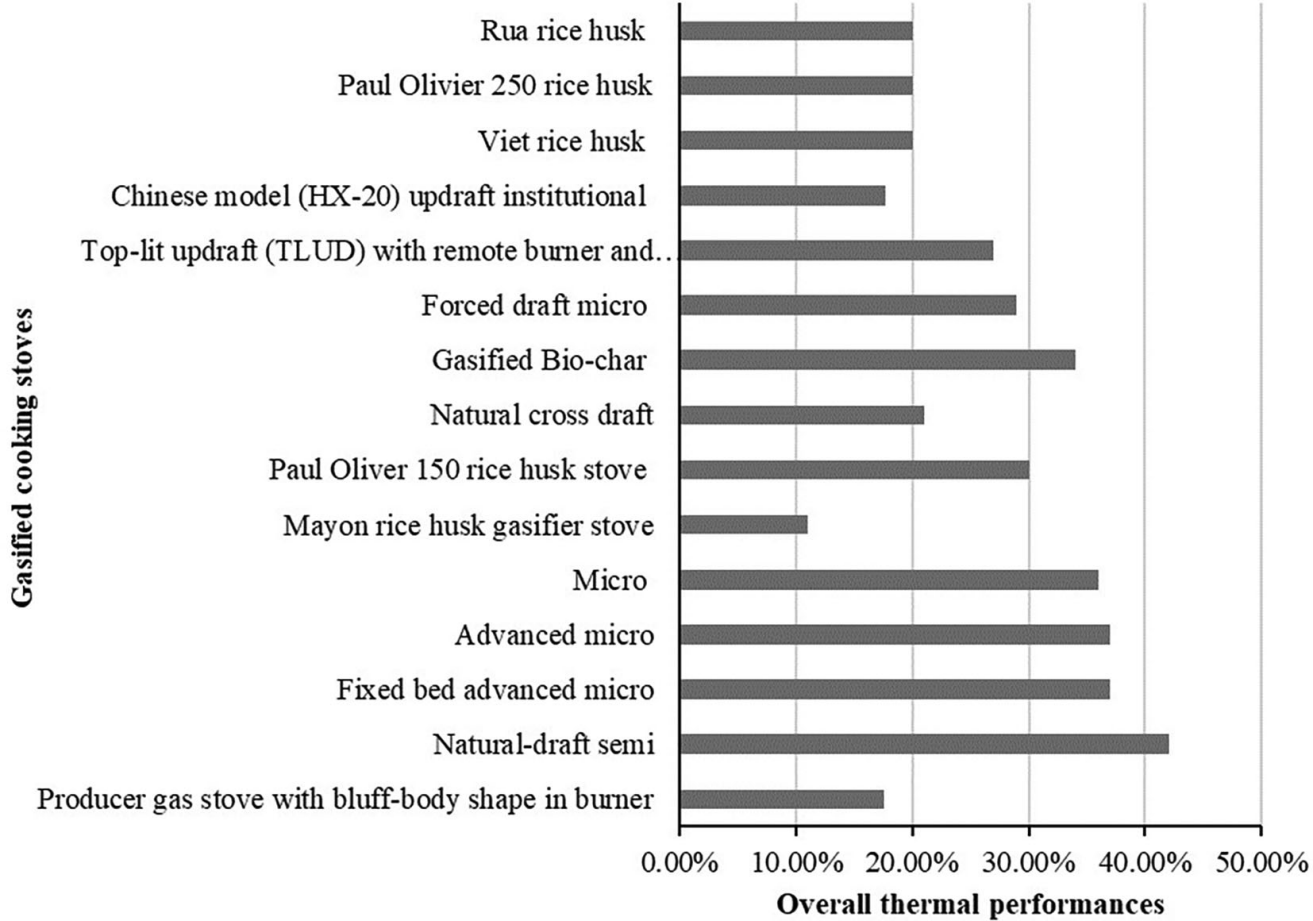
Overall thermal performance of different gasified cooking stoves.

The overall thermal performance of different gasified cooking stoves from Table 2 is identified 5.88% to 91% depends on the design and burning fuels. The thermal performances of the cooking stoves usually determine by using three approaches named water-boiling test, control cooking test and kitchen performance test. The overall thermal performance of different gasified cooking stoves obtained from selected studies is presented in Figure 11 and Table 3. Figure 11 shows that natural draft semi gasified cooking stove provide the highest overall thermal performance which was 42% while Mayon rice husk gasified stoves shows the lowest performance which was 11%. This overall thermal performance of the stoves usually varied due to the design and fuels applied in the experimental tests. In the meantime, the overall thermal performance of some gasified stoves was presented as range in the literature therefore those performance is not presented in Figure 11, which can only find in Table 3. Table 3 shows that premixed producer gas burner with a swirl vane stove provided the highest overall thermal performance range which was 84% to 91% and updraft gasified stove provided the lowest performance which was 5.88% to 8.79%. Swirl vanes use in stoves usually a flame retardant device that highlight the recirculation zone formation to improve the mixing of flame stabilization and reactants compared to other stoves. Due to the improve in flame stabilization and reactants mixture, the performance and efficiency of the stoves are increased compared to other stoves.

**Table 3.** Overall thermal performance range.

Gasified stove types	Overall thermal performance
General gasified	24% to 47%
Premixed producer gas burner with a swirl vane	84% to 91%
Chinese gasifier stove	22 to 33%
Inverted downdraft	10.6% to 38.1%
Top-lit updraft	10.86% to 42%
Natural draft	22.7% to 29.85%
Downdraft	36% to 39%
Forced draft	25% to 36.47%
Forced-draft Chinese three types	16% to 43%
Forced draft with separate secondary and primary air fans	41% to 43%
Updraft	5.88% to 8.79%

**Table 4.** Thermal performances of cooking fuels for gasified cooking stoves.

Cooking Fuel	Overall thermal performance
Coconut shell	36.70%
Prosopis Juliflora	36-37.4%
Wood pellets	38.50%
Tamarind seed pellets	38±0.4%
Eucalyptus	29.85%
Sawdust-cow animal waste briquette	28.43%
Bamboo	23.76%
Peanut shell pellets	31.4±1.2%
Corn cobs	12% to 28%
wood chips	17.76% to 24%
Wood char	17.80%
Rice husk	15.51% to 16.47%
Nut shell pellets	12.38%
Corn straw briquette	10.86%
Pellet	19.91%

### Thermal performances of cooking fuels for gasified cooking stoves

Due to the different mechanical properties such as fuel consumption rate, calorific value, heating rate and fire point different cooking fuels provided different thermal performance presented in Table 2. To understand the insight of the thermal performance of different stoves for different cooking fuels a summarization table is created. The Table 4 presents the overall thermal performance for some cooking fuels that are directly mentioned in Table 2. From Table 4 it can be seen that wood pellets provided the highest thermal performance and corn straw briquette provided the lowest. The overall thermal performance of wood pellets was 38.5% and corn straw briquette was 10.86%. Due to the thermal, physical and biomass characteristics including burning rate, heat capacity, proximate analysis and energy content, wood pellets provided the better perform compared to corn straw briquette.

## Conclusion

In this current literature review the overall thermal performance of different gasified cooking stoves were explored. For this purpose, available literature from past 14 years from 2008 to 2022 were search by using different search strings and after screening a total of 28 articles were selected for this literature review. The key findings from the review are as follows:

• Maximum studies on gasified cooking stoves were conducted on 2019, which was 18%, and the least minimum researches were conducted on 2012, which was only 4%. From the beginning to the mid of the current year 2022 almost 14% studies were identified from the selected literature which reflects that the investigation demand on gasified cooking stoves is recently also in high priority to researcher.
• The identified gasified cooking stoves from literature are classified in six groups named downdraft, updraft, natural draft, forced draft, micro, general gasified and others whereas the maximum articles worked on general gasified cooking stoves, which was 23%.
• 21% published articles on gasified cooking stoves performed their studies in India, which is the highest while the lowest study was performed in Thailand, which was only 3%.
• 15% published articles used mild steel to make gasified stove, which is the highest while only 3% used, ceramic fiber, which is the lowest.
• The identified cooking fuels for gasified stoves are classified in four group which are wooden fuel, animals’ manure, cereals, charcoal and others whereas wooden fuel was applied most of the studies.
• The overall thermal performance of different gasified cooking stoves was 5.88% to 91% depends on the design and burning fuels. The premixed producer gas burner with a swirl vane stove provided the highest overall thermal performance range, which was 84% to 91%, and the updraft gasified stove provided the lowest performance, which was 5.88% to 8.79%.
• Among the coking fuels, the wood pellets provided the highest thermal performance and corn straw briquette provided the lowest for gasified cooking stove. The overall thermal performance of wood pellets was 38.5% and corn straw briquette was 10.86%.

The review recommends to analysis the impact of pollution rate of the identified gasified stove on women and children health. Moreover, the adoption rate among general, economic sustainability and lifecycle analysis of the identified gasified stoves can be more valuable for our community.

## Data Availability

All data underlying the results are available as part of the article and no additional source data are required. Figshare: PRISMA checklist and flowchart for ‘ *Thermal performance of gasifier cooking stoves: A systematic literature review*’, https://doi.org/10.6084/m9.figshare.21747020.v2 ( Uddin *et al.*, 2022). Data are available under the terms of the Creative Commons Zero “No rights reserved” data waiver (CC0 1.0 Public domain dedication).
