# Supplementary material for: Thermal performance of gasifier cooking stoves: A systematic literature review
Source: F1000Res. 2023 Jul 5;12:38. Originally published 2023 Jan 10. [Version 2] doi: 10.12688/f1000research.126890.2 (PMC10357073; doi:10.12688/f1000research.126890.2)
Supplement: Peer review history

# Peer review: Reviewer response for version 2

**DOI:** 10.5256/f1000research.145661.r184648

**Published:** 2023-07-19

**Authors:** Nawshad Arslan Islam Arslan Islam

**Affiliations:** 1 Aerospace Center, The University of Texas at El Paso, El Paso, TX, USA

Satisfied with the revision

Are the rationale for, and objectives of, the Systematic Review clearly stated?

Partly

Is the statistical analysis and its interpretation appropriate?

Not applicable

Are sufficient details of the methods and analysis provided to allow replication by others?

No

Are the conclusions drawn adequately supported by the results presented in the review?

No

Reviewer Expertise:

Advanced Energy Systems, Carbon Negative/Carbon Neutral Hydrogen, Blue Hydrogen, Green Hydrogen, Advanced Power Cycle, Gasification Systems, High speed Flow dynamics, High Pressure Combustion, SCO2 power cycles, High Turbulence Combustion, Digital Engineering, Digital Twin, Smart Grid, Microgrid, V2G, V2L , V2H, V2X, Digitally Interconnected Energy Infrastructure, CFD, FEA. Experimental Thermo-fluids

I confirm that I have read this submission and believe that I have an appropriate level of expertise to confirm that it is of an acceptable scientific standard.

# Peer review: Reviewer response for version 2

**DOI:** 10.5256/f1000research.145661.r184649

**Published:** 2023-07-11

**Authors:** Shabnam Konica

**Affiliations:** 1 Brown University, Providence, RI, USA

Ok to index.

Are the rationale for, and objectives of, the Systematic Review clearly stated?

Yes

Is the statistical analysis and its interpretation appropriate?

I cannot comment. A qualified statistician is required.

Are sufficient details of the methods and analysis provided to allow replication by others?

Partly

Are the conclusions drawn adequately supported by the results presented in the review?

No

Reviewer Expertise:

Mechanical Engineering, Solid Mechanics

I confirm that I have read this submission and believe that I have an appropriate level of expertise to confirm that it is of an acceptable scientific standard.

# Peer review: Reviewer response for version 1

**DOI:** 10.5256/f1000research.139347.r161509

**Published:** 2023-02-10

**Authors:** Nawshad Arslan Islam Arslan Islam

**Affiliations:** 1 Aerospace Center, The University of Texas at El Paso, El Paso, TX, USA

The review focuses on gasifier stoves and their thermal performances. I applaud the hard work of the authors, however there are significant improvements required. 

 Although the literature may be limited due to the technology being limited to use in certain parts of the world,  a wide range of technical resources can be found based on gasification process and biomass power generation. the review fails to merge the scientific relations between such processes and hence does not provide any technical information behind statistical data? For example, the article states wood pellet shows better efficiency but fails to analyze the reason behind it.

 Although a review article, it should necessarily go over adequate amount of technology description. For example the article provides no background on how thermal performances are measured, what experiments are usually performed, examples of such set ups. For a small review of 28 literatures, it was possible to have in depth discussion of some of the technical aspects and design aspects.

 In addition, the review focuses a lot on how the articles were searched and geological location of research and how often research was published. However, I do not think these are relevant and provide any scientific merit to the work.

 I have several recommendations for the authors, if followed will greatly improve the quality of the article. My recommendations are listed below-

1. Abstract should not contain how articles were searched. this is irrelevant for technical paper review. It should focus on how many types of stoves you reviewed, how many fuels (feeds) reviewed, some of the technical challenges, methods used to calculate efficiency and a few line summary of why some setups and fuels perform better than others.
2. In introduction it would be better to talk about biomass fuel types. There is a broader category of fuel types based on source -  Agricultural, Forest, Animal Residue, Solids and Plastic Waste, MSW. And then by constituent of organic matter - Lignin, lignocellulosic, cellulosic, hemi cellulosic etc. Author may want to define the types based such technical norms and then set up the review based on such fuel types. Otherwise the comparisons will not have any common variable. for example woodchip always performs better while agricultural waste will always perform worse due to organic constituent. Thus comparing two different types over several types of stoves will not provide much technical understanding
3. Instead of Dung authors may want to write Animal Waste. It covers a broader area. 
4. The Methods section is completely irrelevant to the technical purpose of the article. If it is not required by the Journal Guideline, it is best to remove this section. 
5. Review papers do not contain Results and Discussion. Since authors are describing what is already published in brief.
6. Instead of Methods and Results and discussion sections, authors may want to have sections as Stove Types -  overviewing different stoves and their design and challenges. Types of Experiments Conducted to measure efficiency. Efficiency by fuel type and how it changes, reasoning behind and how stove design influences it.
7. Similar to the reasons stated above, Figure 1-4, 6 and Table 1 and 2 does not have any meaningful technical contribution and thus should be removed if not required by Journal guideline. 
8. In addition, the data for figure 2-6 are questionable. Since the author filters a lot of literatures, such numbers do not reflect actual scenario. Thus it is best to avoid it. 
9. There is a tremendous scope to add technical reasoning for this review. For example, each type of cooking stove efficiency should be backed up by how it was measured and what setup was used and how to calculate it.
10. Similar reasoning should also be used to describe efficiency issues for fuels
11. Why was swirl vane producer gas burner was not included in gasified stoves comparison in figure 7. Essentially Gasification or Gasified process results in producer gas?
12. Why does the premixed producer gas with swirl vane have a very high efficiency compared to other technologies? Please describe
13. Similarly why does wood pellets perform better than corn straw? Please describe
14. Similar descriptions should be provided for all cases  presented in Table 3, 4 and 5

I wish the authors best of luck with their future work.

Are the rationale for, and objectives of, the Systematic Review clearly stated?

Partly

Is the statistical analysis and its interpretation appropriate?

Not applicable

Are sufficient details of the methods and analysis provided to allow replication by others?

No

Are the conclusions drawn adequately supported by the results presented in the review?

No

Reviewer Expertise:

Advanced Energy Systems, Carbon Negative/Carbon Neutral Hydrogen, Blue Hydrogen, Green Hydrogen, Advanced Power Cycle, Gasification Systems, High speed Flow dynamics, High Pressure Combustion, SCO2 power cycles, High Turbulence Combustion, Digital Engineering, Digital Twin, Smart Grid, Microgrid, V2G, V2L , V2H, V2X, Digitally Interconnected Energy Infrastructure, CFD, FEA. Experimental Thermo-fluids

I confirm that I have read this submission and believe that I have an appropriate level of expertise to confirm that it is of an acceptable scientific standard, however I have significant reservations, as outlined above.

# Response

**Published:** 2023-04-17

**Authors:** Md Insiat Islam Rabby

**Affiliations:** Milittary Institute of Science and Technology, Dhaka, Bangladesh, Bangladesh

Thank you very much for reviewing the manuscript. The paper has been revised according to the reviewer suggestions and comments. The responses for reviewer comments are as follows:

 1. Abstract should not contain how articles were searched. this is irrelevant for technical paper review. It should focus on how many types of stoves you reviewed, how many fuels (feeds) reviewed, some of the technical challenges, methods used to calculate efficiency and a few line summary of why some setups and fuels perform better than others.

**Response:** Thank you for the concern. This is actually a systematic literature review paper not a technical review paper. Therefore, reviewer might me confused on about the current review. Authors maintained PRISMA systematic literature review process, which was provided by the journal to make a good systematic literature review. In published other systematic review papers it is common to mention how articles were searched and how many articles were selected for review.

 However, authors added the number of stoves and fuels identified from literature in the Abstract section.

 2. In introduction it would be better to talk about biomass fuel types. There is a broader category of fuel types based on source -  Agricultural, Forest, Animal Residue, Solids and Plastic Waste, MSW. And then by constituent of organic matter - Lignin, lignocellulosic, cellulosic, hemi cellulosic etc. Author may want to define the types based such technical norms and then set up the review based on such fuel types. Otherwise the comparisons will not have any common variable. for example woodchip always performs better while agricultural waste will always perform worse due to organic constituent. Thus comparing two different types over several types of stoves will not provide much technical understanding

**Response:** Thank you for the nice suggestion. As this study, mainly focus on revising gasified cooking stoves and its thermal performance therefore in the introduction section talk about fuel types is beyond the scope of the review. The main focus of the literature review is gasified cooking stoves and thermal performance not fuel types. Even those published articles we reviewed from literature they also did not discuss on fuel types and physical characteristics of fuels. Therefore, authors want to keep the arrangement of introduction section as it is. However, authors added working principal and design of different gasified cooking stoves in results and discussion section to make the review more standard.

 3. Instead of Dung authors may want to write Animal Waste. It covers a broader area. 

**Response:** Thank you so much for the suggestion. Authors changed dung to animal waste.

 4. The Methods section is completely irrelevant to the technical purpose of the article. If it is not required by the Journal Guideline, it is best to remove this section. 

**Response:** This is actually a systematic literature review paper not a technical review paper. Therefore, reviewer might me confused on about the current review. Authors maintained PRISMA systematic literature review process, which was provided by the journal to make a good systematic literature review. In published all systematic review papers reviewer can easily find method section. It is a mandatory part for systematic literature review  according to PRISMA guideline. Therefore, authors added this section otherwise it will not be a systematic review.

 5. Review papers do not contain Results and Discussion. Since authors are describing what is already published in brief.

**Response:** This is actually a systematic literature review paper not a technical review paper. Therefore, reviewer might me confused on about the current review. Authors maintained PRISMA systematic literature review process, which was provided by the journal to make a good systematic literature review. In published all systematic review papers reviewer can easily find result and discussion section. It is a mandatory part for systematic literature review according to PRISMA guideline. Therefore, authors added this section otherwise it will not be a systematic review.

 6. Instead of Methods and Results and discussion sections, authors may want to have sections as Stove Types -  overviewing different stoves and their design and challenges. Types of Experiments Conducted to measure efficiency. Efficiency by fuel type and how it changes, reasoning behind and how stove design influences it.

**Response:** This is actually a systematic literature review paper, not a technical review paper. Therefore, the reviewer might be confused about the current review. Authors maintained PRISMA systematic literature review process, which was provided by the journal to make a good systematic literature review. In published all systematic review papers reviewer can easily find result and discussion sections. It is a mandatory part for systematic literature review according to PRISMA guidelines. Therefore, authors added this section otherwise it will not be a systematic review.

 However, the authors added a subsection on different cooking stoves according to reviewer's suggestion where design and working principles were discussed.

 7. Similar to the reasons stated above, Figure 1-4, 6 and Table 1 and 2 does not have any meaningful technical contribution and thus should be removed if not required by Journal guideline. 

**Responses:** Authors removed Table 2 and Figure 3. The authors want to keep others' figures and tables to maintain the systematic review protocol.

 8. In addition, the data for Figure 2-6 are questionable. Since the author filters a lot of literature, such numbers do not reflect the actual scenario. Thus it is best to avoid it. 

**Responses:** Authors want to keep these figure and tables to maintain systematic review protocol. As the search and article selection protocol maintained a systematic method therefore the presented scenario is actual.

 9. There is a tremendous scope to add technical reasoning for this review. For example, each type of cooking stove efficiency should be backed up by how it was measured and what setup was used and how to calculate it.

**Response:** The measurement process of efficiency and setup were not scope of the review. Authors working on a general technical review on cooking stoves where these will be added.

 10. Similar reasoning should also be used to describe efficiency issues for fuels

**Response:** The measurement process of efficiency and setup was not the scope of the review. Authors working on a general technical review on cooking stoves where these will be added.

 11. Why was swirl vane producer gas burner was not included in gasified stoves comparison in figure 7. Essentially Gasification or Gasified process results in producer gas?

**Responses:** Thank you for the comment. The performance of  swirl vane producer gas burner was identified as range (84% to 91% ) in literature therefore it was not included in Figure 7. In Figure 7, only those stoves' performance that was not in range was plotted. However, in discussion, the performance of swirl vane producer gas burner is included.

 12. Why does the premixed producer gas with swirl vane have a very high efficiency compared to other technologies? Please describe

**Response: **Thank you for the suggestions. The reason has been included in “Thermal performance of different gasified cooking stoves” section. The added part is as follows:

 “Swirl vanes use in stoves are usually a flame retardant device that highlights the recirculation zone formation to improve the mixing of flame stabilization and reactants compared to other stoves. Due to the improvement in flame stabilization and reactants mixture, the performance and efficiency of the stoves are increased compared to other stoves”

 13. Similarly why do wood pellets perform better than corn straw? Please describe

**Response:** Thank you for the suggestions. The reason has been included in the “Thermal performances of cooking fuels for gasified cooking stoves” section.

 14. Similar descriptions should be provided for all cases  presented in Tables 3, 4 and 5

**Response: **Short description has been provided for all cases  presented in Tables 3, 4 and 5

# Peer review: Reviewer response for version 1

**DOI:** 10.5256/f1000research.139347.r161508

**Published:** 2023-02-07

**Authors:** Shabnam Konica

**Affiliations:** 1 Brown University, Providence, RI, USA

This work presents a statistical review of the thermal performance of gasifier cooking stoves. While the content of this article is important to understand the aspects of designing and energy performance evaluation of gas stoves for the countries where this type of stoves are still in use, it does not have a clear conclusion or future recommendations regarding how to improve the performance in terms of energy efficiency and environmental pollution. There should be more concise action words to address this issue. Below are some of my concerns:

1. I am against putting Table-2 altogether. Everyone knows how to search, and it should not be on the table. The references are included, which should suffice the explanation of the search method.
2. What are the authors' conclusions about the different types of cooking stoves? Which design is better and why? More explanations are necessary. Also, I advise including their design and working principles.
3. The authors mention, "this finding will help researchers, organizations and government to investigate and implement this type of cooking stoves based on the geographical location so that the adoption rate of the research can increase." If these stoves are already in use in those locations, it is unclear why the government should implement them. They are already in use.
4. The authors talk about different materials. While it is clear why steel stoves are widely used for fabricating gas stoves, I am not clear why other materials are there. Do those materials improve the design and efficiency? More explanation is necessary.
5. For each fuel, there should be sufficient research on the health impact and environmental pollution. The authors should conduct a review in these areas and add it to this work, and it should not be left for future recommendations. 

Are the rationale for, and objectives of, the Systematic Review clearly stated?

Yes

Is the statistical analysis and its interpretation appropriate?

I cannot comment. A qualified statistician is required.

Are sufficient details of the methods and analysis provided to allow replication by others?

Partly

Are the conclusions drawn adequately supported by the results presented in the review?

No

Reviewer Expertise:

Mechanical Engineering, Solid Mechanics

I confirm that I have read this submission and believe that I have an appropriate level of expertise to state that I do not consider it to be of an acceptable scientific standard, for reasons outlined above.

# Response

**Published:** 2023-04-17

**Authors:** Md Insiat Islam Rabby

**Affiliations:** Milittary Institute of Science and Technology, Dhaka, Bangladesh, Bangladesh

Thank you very much for reviewing the manuscript. The paper has been revised according to the reviewer suggestions and comments. The responses for reviewer comments are as follows:

 1. I am against putting Table-2 altogether. Everyone knows how to search, and it should not be on the table. The references are included, which should suffice the explanation of the search method.

**Response:** Thank you for the suggestion. The table has been removed.

 2. What are the authors' conclusions about the different types of cooking stoves? Which design is better and why? More explanations are necessary. Also, I advise including their design and working principles.

**Response:** Thank you for the suggestion and advice. Authors added the design, working principal and reason of performance for different identified gas stoves.

 3. The authors mention, "this finding will help researchers, organizations and government to investigate and implement this type of cooking stoves based on the geographical location so that the adoption rate of the research can increase." If these stoves are already in use in those locations, it is unclear why the government should implement them. They are already in use.

**Response:** This review only summarized the articles those are experimentally conducted in laboratory by different researchers and identified thermal performances. Authors did not get any evidence from the literature that those are implanted and used in big scale in those geographical areas therefore authors mentioned that statement based on the findings from literature. However, for readers continence authors made the following revisions:

 “this finding may help researchers, organizations and government to investigate and implement this type of cooking stoves based on the geographical location so that the adoption rate of the research can increase”  

 4. The authors talk about different materials. While it is clear why steel stoves are widely used for fabricating gas stoves, I am not clear why other materials are there. Do those materials improve the design and efficiency? More explanation is necessary.

**Response:** Thank you for looking on it. During discussing materials authors missed some points therefore this section seems unclear. For manufacturing stoves steel was used in all studies, while other mentioned materials usually used for insulation purposes. Authors revised the “Materials to fabricate stoves” section accordingly.

 5. For each fuel, there should be sufficient research on the health impact and environmental pollution. The authors should conduct a review in these areas and add it to this work, and it should not be left for future recommendations.

**Response:** Thank you for the valuable suggestion. Authors also believe that health impact and environmental pollution are very concerning factors for cooking stoves. However, the current systematic literature review only limited to thermal performance of gasifier cooking stoves. Health impact and environmental pollution are not scope of this review. Therefore, authors only showed the relationship between fuels and thermal performances for gasified cooking stoves. This is a systematic literature review, which was structured and maintained according to the guidelines of a typical systematic review followed by “PRISMA Diagram & Checklist - Systematic Reviews”. Additionally, most of the identified literature for this review did not discuss health impact and environmental pollution are not scopes of this review. Therefore, it will be now very difficult for authors to add health impact and environmental pollution, which were not the scopes for this review. Authors are now writing another critical review article on cooking stoves wherein health impact and environmental pollution are considered and given priority, therefore authors do not want to include health impact and environmental pollution in this systematic review.
